# Litchi LcAP1-1 and LcAP1-2 Exhibit Different Roles in Flowering Time

**DOI:** 10.3390/plants14172697

**Published:** 2025-08-29

**Authors:** Qiulin Gui, Jinju Wei, Ziang Wu, Xiao Mo, Haowei Qing, Yuyu Shi, Huiqin Guo, Jingwen Sheng, Feng Ding, Shuwei Zhang

**Affiliations:** 1 Guangxi Key Laboratory of Genetic Improvement of Crops, Guangxi Academy of Agricultural Sciences, Nanning 530007, China; qt15797862018@163.com (J.W.); 18376683951@163.com (X.M.); huigin@163.com (H.G.); semniwer@sina.com (J.S.); 2 College of Agriculture, State Key Laboratory for Conservation and Utilization of Subtropical Agro-Bioresources, Guangxi University, Nanning 530004, China; gql19898@163.com (Q.G.); wza67690@163.com (Z.W.); 18777200815@163.com (H.Q.); sy1045731981@163.com (Y.S.); 3 Horticultural Research Institute, Guangxi Academy of Agricultural Sciences, Nanning 530007, China

**Keywords:** litchi, flowering, *AP1* homologous gene, expression analysis, transgenic tobacco

## Abstract

Litchi (*Litchi chinensis* Sonn.) is a kind of evergreen fruit tree with good flavor and taste which has high economic value. Sufficiently low temperature in winter is essential for the successful flower formation of litchi. Therefore, in the context of global warming, litchi often experiences unstable flower formation, ultimately resulting in a decrease in litchi production. Our previous research has highlighted the pivotal role of the *LcFT1* gene in regulating the flower formation of litchi and identified two *AP1* homologous genes associated with *LcFT1* (named *LcAP1-1* and *LcAP1-2*) based on RNA-Seq and weight gene co-expression network analysis (WGCNA). In this study, the functions of the two *AP1* homologous genes in regulating flowering time were investigated. Result showed that *LcAP1-1* and *LcAP1-2* were expressed in all litchi tissues. *LcAP1-1* was more highly expressed in mature leaves compared to other tissues, while the *LcAP1-2* has the highest expression level in flower buds. Both of them exhibited upregulation in the terminal bud of litchi under low temperature. The expression of *LcAP1-1* and *LcAP1-2* was highly correlated with the initiation of flower buds and the development of flower organs. They increased gradually during the floral initiation but decreased gradually during flower bud development. The transgenic tobacco of *LcAP1-2* flowered about 55 days earlier than wild-type, while tobacco overexpressing the *LcAP1-1* gene had no significant changes in flowering time compared to the wild-type. These results indicate that the two genes have divergent regulatory functions, and that the *LcAP1-2* gene may be involved in the regulation of flower transformation and flower organ development in litchi. Our research will further reveal the molecular regulatory mechanisms of flower formation in litchi and will also provide theoretical guidance for the molecular breeding of litchi.

## 1. Introduction

In plants, flowering is a sign of the transition from vegetative growth to reproductive growth and a necessary process for successful plant reproduction. It generally includes four stages, including flower induction, inflorescence meristem formation, flower initiation, and flower organ morphogenesis. The regulation of flowering transition involves a complex genetic regulatory network control process [1,2]. Currently, flowering is understood to be regulated by photoperiodic pathway, vernalization pathway, autonomic pathway, gibberellin pathway, thermosensitive pathway, and age pathway [3]. The signals directly or indirectly activate the expression of *FLOWERING LOCUS T* (*FT*), *LEAFY* (*LFY*), and *APETALA1* (*AP1*) genes through the photoperiodic pathway, gibberellin pathway, and age pathway to initiate flowering. The *AP1* homologous gene belongs to the MADS-box family of transcription factors. The isolation and analysis of numerous MADS-box genes in plants has revealed that the majority of this type of transcription factor are intricately involved in the process of flower development [4]. MADS-box includes the MADS region, K region, I region, and C region. The MADS region is essential for DNA binding and protein dimerization, and it is also the most conserved region between *AP1* and its homologous genes. The C region performs a transcriptional activation function [5].

In the ABC (DE) flower development model, *AP1* is a class A gene. It is a floral primordium response characteristic gene that regulates floral meristem differentiation and the formation of calyxes and petals [6,7]. It is a gene downstream in the flowering regulatory network, involved in multiple flowering pathways, and has relationship with *AP1* and many genes related to flowering regulation [8,9,10,11]. The *AP1* homologous genes coordinate with *APETALA2* (*AP2*), *APETALA3* (*AP3*), *PISTILLATA* (*PI*), and *AGAMOUS* (*AG*) to regulate the differentiation of floral organs [12]. A complex of FT and FD proteins can activate transcription factors *AP1, SOC1, FRUITFULL (FUL),* and *SEPALLATA3 (SEP3)* to regulate the transition from vegetative growth to reproductive development in plants [13]. The expression of *AP1* is closely associated with *LFY*. *AP1* and *LFY* are important genes involved in the transition from vegetative to reproductive growth, and the *LFY* induces the early expression of *AP1* [14]. *AP1* and *LFY* interact and regulate flower development during floral initiation [15]. Moreover, *LFY* can induce the expression of gibberellin catabolic genes. Increased *LFY* activity consequently reduces gibberellin levels, resulting in the accumulation of gibberellin-sensitive DELLA proteins. This accumulation promotes the upregulation of *AP1*, which in turn facilitates flowering in Arabidopsis [16]. *AP1* and *LFY* are activated by the transcription factor *MdNF-YC2* to initiate flowering [17].

So far, *AP1* homologous genes have been isolated from various woody plants, and extensive research has been conducted on their expression and functionality. *ZjAP1* in jujube plays a role in both vegetative and reproductive development [18]. The transcription of *EjAP1* was detected in the inflorescence buds and flower tissues of loquat, but not in the vegetative tissues [19]. *EjAP1* was significantly up-regulated in loquat at the stages of flower bud differentiation [20]. *AP1* isolates from peach were expressed specifically in the flower [21]. After low-temperature treatment, the expression level of *VcAP1* was up-regulated in blueberry buds [22]. Transgenic analysis demonstrated that the overexpression of *AP1* genes can promote flowering in transgenic plants [23,24,25,26,27,28]. Inducing *AP1* overexpression through exogenous substance application regulates plant flower bud differentiation and flowering time. For example, the expression of the *AP1* gene was induced by the application of H_2_O_2_, KClO_3_, and NO in order to regulate flowering in off-season longan [29,30]. Similarly, the application of potassium nitrate and ethephon promoted mango flower bud differentiation, and the upregulation of *AP1* was observed [31].

Litchi (*Litchi chinensis* Sonn.), originating in China, is now cultivated in 20 countries including Australia, South Africa, and Vietnam. As a significant subtropical evergreen fruit crop, its production value constitutes an important economic resource for local regions [2,32]. In litchi cultivation, unstable flowering directly leads to reduced yields. Moreover, inadequate flowering triggers the “off-year” phenomenon [13,33]. Additionally, the concentrated harvest period coupled with its perishable nature results in market gluts, often causing a “bumper harvest without proportional profits” scenario that severely impedes economic efficiency improvements [2].

Flowering is a critical factor influencing litchi yield and fruit ripening timing. The transcription factor *AP1* plays an important role in regulating flowering. Once activated, *AP1* functions as a flowering promoter in plants [17]. Our previous research has emphasized the key role of the *LcFT1* gene in regulating litchi flowering. Based on RNA Seq technology and using weighted gene co-expression network (WGCNA) analysis, we found that two *AP1* homologous genes (*LcAP1-1* and *LcAP1-2*) were strongly associated with the *LcFT1* gene, suggesting that *LcFT1* regulates *LcAP1* overexpression and litchi flowering [13]. It is speculated that *LcFT1* activates downstream transcription factor *LcAP1* overexpression, leading to litchi flowering. It is necessary to further investigate the specific impact of *LcAP1-1* and *LcAP1-2* on litchi flowering. Therefore, this study aims to investigate the regulatory roles of *LcAP1-1* and *LcAP1-2* in litchi flowering time through spatiotemporal expression profiling of *LcAP1-1* and *LcAP1-2* functional validation in transgenic plants, elucidating the relevant molecular mechanisms. This will help improve the flowering and development process of litchi using molecular breeding methods, provide theoretical basis for cultivating litchi varieties at different maturity stages, and identify key target genes for litchi molecular breeding.

## 2. Result

### 2.1. Isolation and Phylogenetic Analysis of LcAP1-1 and LcAP1-2 Genes in Litchi

**Figure 1:**
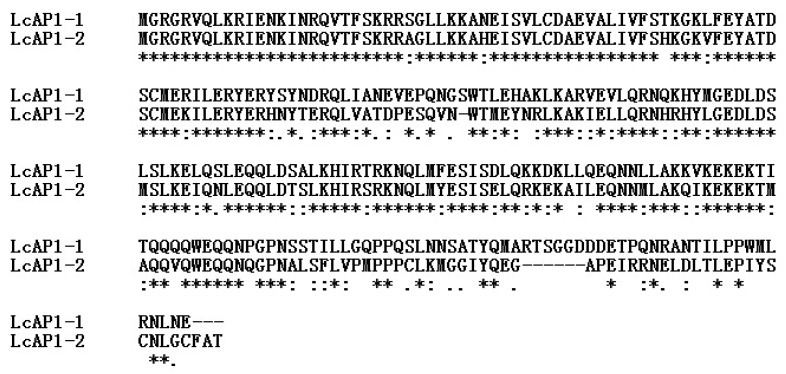
Alignment of the amino acid sequences of litchi LcAP1-1 and LcAP1-2 proteins. The asterisk (*) denotes identical sequence residues.

**Figure 2:**
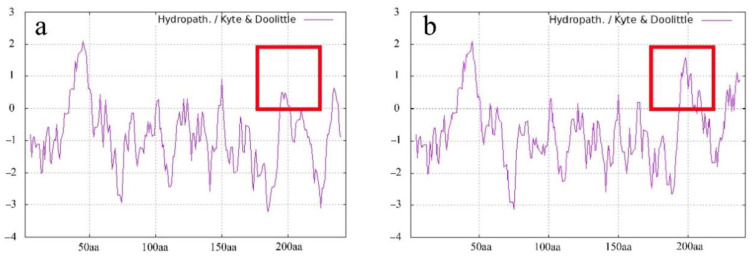
Hydrophilic or hydrophobic peaks of LcAP1-1 and LcAP1-2 proteins. (**a**) Peak hydrophilicity of LcAP1-1 protein. (**b**) Peak hydrophilicity of LcAP1-2 protein. The red box indicates the difference in hydrophilicity between the LcAP1-1 and LcAP1-2 proteins.

**Figure 3:**
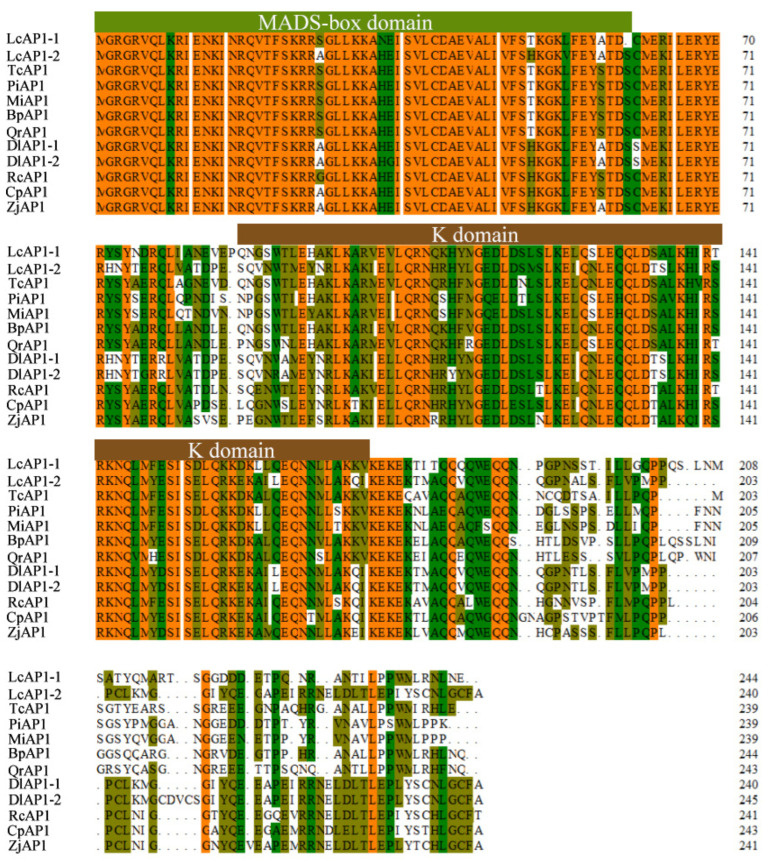
Alignment of the predicted amino acid sequences of LcAP1-1 and LcAP1-2 and other AP1 homologous proteins from other woody plants. The MADS-box domain and K-box domain are shown in the boxes above the aligned sequences. Multiple alignment of the *AP1* proteins from *Theobroma cacao* (TcAP1, EOY22135.1), *Pistacia integerrima* (PiAP1, KAJ0047719.1), *Mangifera indica* (MiAP1, URM60843.1), *Betula pendula* (BpAP1, CAA67969.1), *Quercus robur* (QrAP1, XP_050257465.1), *Dimocarpus longan* (DlAP1-1, AEZ63951.1, DlAP1-2, AGC13077.1), *Ricinus communis* (RcAP1, XP_002512051.2), *Carica papaya* (CpAP1, XP_021910321.1), and *Ziziphus jujuba var. spinosa* (ZjAP1, XP_048317859.1). Yellow indicates that the sequences are completely identical, dark green indicates similarity ≥ 75%, and light green indicates similarity ≥ 50%.

**Figure 4:**
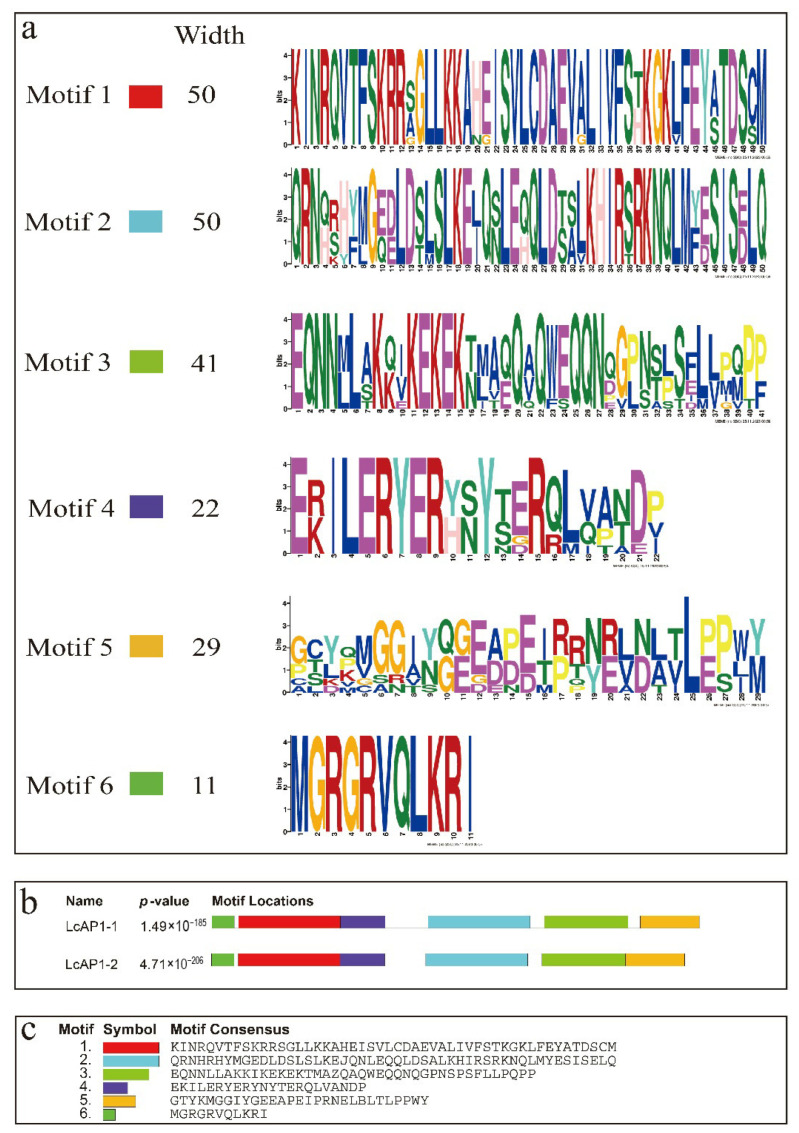
Conserved motifs of LcAP1-1 and LcAP1-2 proteins. (**a**) Logo of the six motifs. (**b**) The location of the Motifs at LcAP1-1 and LcAP1-2. (**c**) Conserved amino acid sequence.

**Figure 5:**
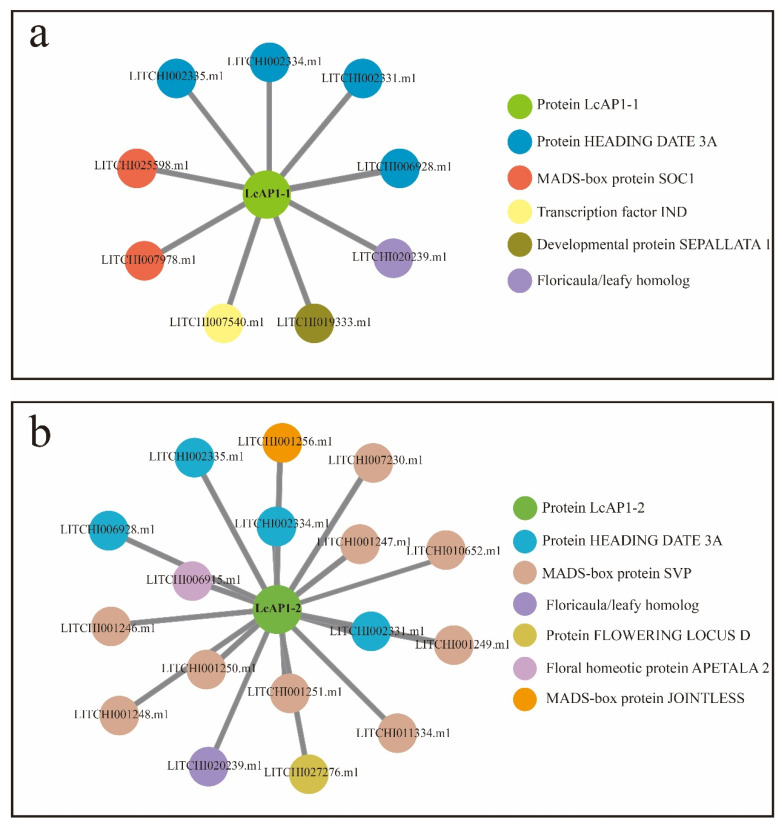
The predicted interaction proteins of LcAP1-1 and LcAP1-2 proteins. (**a**) LcAP1-1 interacted with nine proteins. (**b**) LcAP1-2 protein interacted with 17 Proteins.

*LcAP1-1* and *LcAP1-2* were isolated with an open reading frame (ORF) length of 738 bp and 726 bp, which encoded proteins of 246 and 242 aa residues, respectively. A comparison of LcAP1-1 and LcAP1-2 predicted proteins showed that their N-terminus is highly conserved, while their C-terminus is significantly different (Figure 1), implying potential functional divergence. Both LcAP1-1 and LcAP1-2 proteins contain 20 amino acids, none of which contains a transmembrane domain, and both belong to unstable proteins. In the hydrophilicity map of LcAP1-1 protein and LcAP1-2 protein, positive values indicate hydrophobic regions, while negative values indicate hydrophilic regions. The hydrophilicity of LcAP1-1 protein and LcAP1-2 protein shows a large difference around 200 aa length (Figure 2). The grand average hydrophilicity and isoelectric point of LcAP1-1 were 0.889 and 9.21, respectively. The two protein sequences contained a MADS-box domain and a K-box domain (Figure 3). The protein motifs (conserved motifs) of two homologous sequences of LcAP1 were analyzed using the online database MEME, and six motifs with lengths ranging from 11 to 50 aa were obtained (Figure 4a). Both LcAP1-1 and LcAP1-2 contain these six motifs (Figure 4b). Motif 1 and Motif 6 belong to conserved motifs of the MADS-box gene family. Their sequences were MGRGRVQLKRI and KINRQVTFSKRR SG LLKKAHEISVLCDAEVALIVFSTKGKLFEYATDSCM, respectively (Figure 4c). These results suggest that *LcAP1-1* and *LcAP1-2* belong to the MADS-box gene family. Using the litchi genome database, the interacting proteins of LcAP1 were predicted, and it was found that LcAP1-1 interacted with HEADING DATE 3A, MADS-box protein SOC1, transcription factor IND, developmental protein SEPALLATA 1, and Floricaula/LEAFY homolog (Figure 5a). The LcAP1-1 protein interacted with protein HEADING DATE 3A, MADS-box protein SVP, Floricaula/leafy homolog, protein FLOWERING LOCUS D, Floral homeotic protein APETALA2, and MADS-box protein JOINTLESS (Figure 5b).

**Figure 6:**
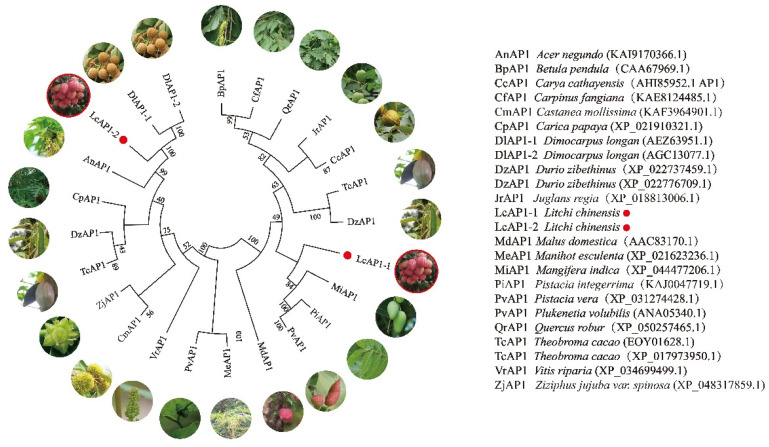
The phylogenetic tree of LcAP1 and other plant AP1 proteins. This tree was created using the neighbor-joining method and multiple sequence alignment with MEGA11. Red dots indicate the AP1 protein on litchi.

A phylogenetic tree of LcAP1 with other species was generated via MEGA11 software using the neighbor-joining method (Figure 6). The results showed that LcAP1-2 has a close evolutionary relationship to *Dimocarpus longan* DlAP1. However, LcAP1-1 has a close evolutionary relationship with *Mangifera indica* MiAP1, which indicates that LcAP1-1 and LcAP1-2 may have divergent functions. The degree of homology among AP1 in these species essentially corresponds to their evolutionary relationship distance. LcAP1-1 is classified into the same group as the *Mangifera indica* family and the *Pistacia integerrima* family. Litchi and Longan both belong to the sapindaceae family; LcAP1-2 was categorized into the same group as longan AP1-1 and AP1-2. The results suggest that there is a differentiation of the *AP1* gene among woody and non-woody plants, as well as within different families and genera, and even within the same species.

### 2.2. Expression Pattern Analysis of LcAP1-1 and LcAP1-2 Genes in Litchi

**Figure 7:**
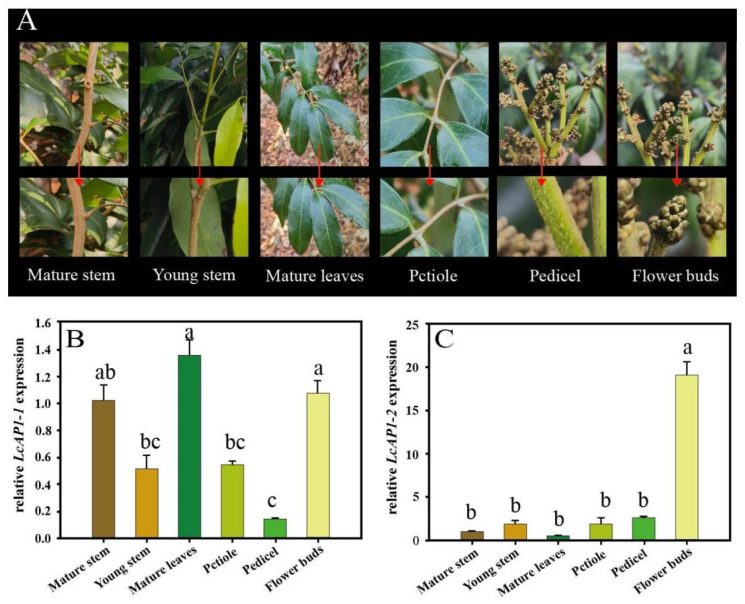
Relative expression level of *LcAP1-1* and *LcAP1-2* genes. (**A**) Different tissues of litchi mature stem, young stem, mature leaves, petiole, pedicel, and flower buds. The arrow indicates the sampling location. (**B**) Expression patterns of *LcAP1-1* gene in different tissues. (**C**) Expression of *LcAP1-2* gene in different tissues of litchi. *LcActin* gene was selected as a reference gene. Data are means from three biological replicates ± SE (*n* = 3). Different letters meant significant difference among the samples (*p* < 0.05).

Quantitative real-time PCR (qRT-PCR) was used to analyze the expression patterns of *LcAP1-1* and *LcAP1-2* genes in litchi tissues. In different organizations (Figure 7A), the results demonstrated ubiquitous expression of the *LcAP1-1* gene in all tested tissues, with a high expression level observed in mature stems and flower buds, and the highest expression level observed in mature leaves (Figure 7B). The expression of the *LcAP1-2* gene was observed in all tissues; however, its expression level in floral buds was seen to be significantly higher compared to other tissues, with a difference of about eight to nine times higher (Figure 7C). These results demonstrated that the functions of the two genes may be different, and that the *LcAP1-2* gene may be involved in the process of flower development.

**Figure 8:**
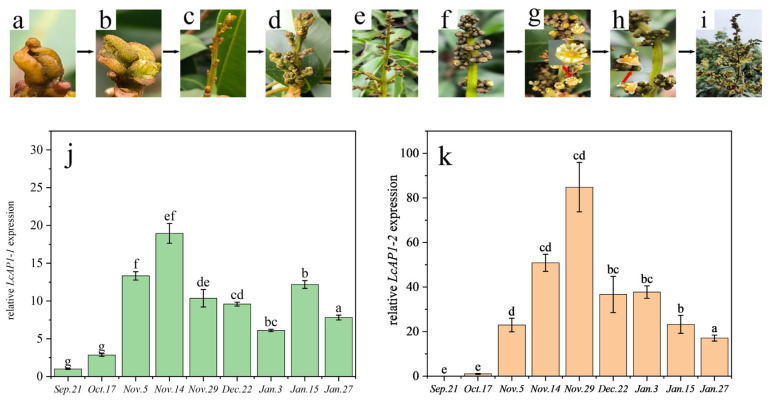
Expression levels of *LcAP1-1* and *LcAP1-2* at the differentiation stage of flower buds. (**a**–**i**) Morphological characteristics of terminal buds of ‘Sanyuehong’ litchi. The relative expression analysis of *LcAP1-1* (**j**) and *LcAP1-2* (**k**) genes using quantitative RT-PCR. Data are means from three biological replicates ± SE (*n* = 3). Different letters meant significant difference among the samples (*p* < 0.05).

We collected the terminal buds throughout the entire reproductive growth stage of litchi floral transition, including before and after low-temperature induction (Figure 8a–i), and examined the expression levels of the *LcAP1-1* and *LcAP1-2* genes. The results suggest a strong correlation between the expression levels of the *LcAP1-1* and *LcAP1-2* genes and flower bud initiation, as well as flower organ development, in litchi. On the whole, the expression levels of the *LcAP1-1* and *LcAP1-2* genes gradually increased during flower bud initiation and the development of flower organs in litchi. However, the expression levels subsequently declined after the completion of flower organ development (Figure 8j,k). Interestingly, the *LcAP1-2* gene was hardly detectable prior to low temperature induction, but it exhibited a rapid and significant increase after low-temperature induction, reaching levels ten times higher than before. Subsequently, the expression of *LcAP1-2* gene decreased rapidly after the completion of flower organ development in litchi. The results indicate that the *LcAP1-2* gene is more strongly associated with flower bud initiation and flower organ development in litchi compared to the *LcAP1-1* gene.

### 2.3. LcAP1-1 and LcAP1-2 Genes Transformation in Tobacco

**Figure 9:**
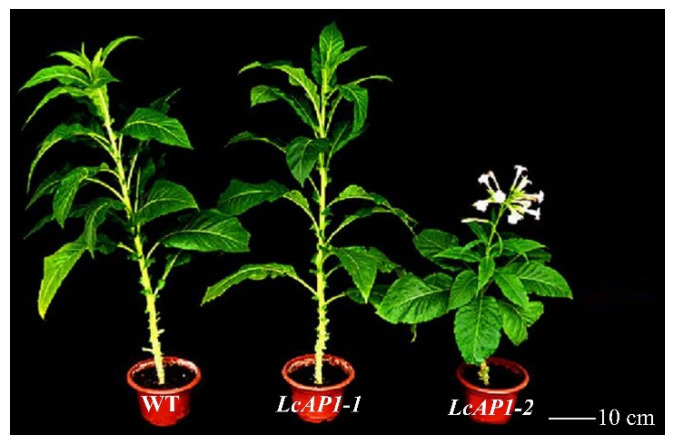
*LcAP1-1* and *LcAP1-2* transgenic tobaccos and comparison of flowering phenotypes.

To further investigate the function of *LcAP1-1* and *LcAP1-2*, the coding sequence was cloned into a pBI121 vector driven by the CaMV35S promoter. Finally, we successfully obtained a total of 28 T0 transgenic tobacco plants overexpressing *LcAP1-1* and 42 T0 transgenic tobacco plants overexpressing *LcAP1-2*. Surprisingly, transgenic tobacco was grown to T3 generation with stable traits, the *LcAP1-2* transgenic tobacco plants showed an obvious early flowering phenotype compared with the wild type (Figure 9). The transgenic plants overexpressing *LcAP1-2* (OE- *LcAP1-2*) exhibited a flowering time of approximately 80 days, whereas the wild-type (WT) tobacco required about 135 days to initiate flowering. In contrast, no significant changes in the flowering time were observed in the transgenic plants overexpressing *LcAP1-1* (OE-*LcAP1-1*). Notably, *LcAP1-2* transgenic tobacco showed obvious dwarf sizing compared with wild-type tobacco (Figure 9), whereas no obvious change in plant height was observed in these plants overexpressing *LcAP1-1*. The findings suggested divergent functionalities between the two genes, with the *LcAP1-2* gene potentially assuming a pivotal role in floral transformation and the development of floral organs in litchi. *LcAP1-2* may also affect vegetative growth and control plant height.

By counting the phenotypic traits of transgenic tobacco plants at flowering (Table 1), it was found that the flowering time of *LcAP1-2* transgenic tobacco plants was significantly earlier than that of *LcAP1-1* and wild-type tobacco plants. In terms of plant height at flowering, the height of *LcAP1-2* transgenic tobacco was about 42 cm, which was half that of *LcAP1-1* and wild-type tobacco plants, while there was no significant difference in height between wild-type and *LcAP1-1* transgenic tobacco plants. At flowering, *LcAP1-1* and wild-type tobacco had about 24 and 22 leaves, respectively, and *LcAP1-2* transgenic tobacco had 14 leaves, which was significantly less than that of the wild-type and *LcAP1-1* transgenic tobacco.

## 3. Discussion

The various pathways regulating plant flowering are relatively independent and influence each other and finally combine into a complex and precise flowering regulation network. The *AP1* gene regulates the plant flowering downstream *FT* gene. The flowering time is one of the key factors affecting the ripening stage of litchi fruit. In production, litchi often suffers from low prices due to the overly concentrated ripening period, which seriously affects the improvement of the economic benefits of the litchi industry. Conducting research on the molecular regulatory mechanisms of litchi flowering time will provide a theoretical basis for the future regulation of litchi ripening periods. Therefore, it is of great research significance. In this study, we cloned and isolated two *AP1* homologous genes from ‘Sanyuehong’ litchi, named *LcAP1-1* and *LcAP1-2*, respectively. Both of them have a MADS-box domain and a K-box domain. Phylogenetic tree analysis indicated that *LcAP1-2* was very closely related to logan *AP1-1*. However, *LcAP1-1* exhibited an extensive evolutionary relationship with other *AP1* genes. The above results indicate that the *LcAP1-1* and *LcAP1-2* genes in litchi may play different roles in the regulation of flowering, which is consistent with the differences in their functions.

In *Arabidopsis thaliana*, *FT* promotes flowering as part of a florigen activation complex (FAC) with the *bZIP* transcription factor *FLOWERING LOCUS D* (*FLD*). However, *AP1* and *LFY*, as genes encoding flowering-promoting proteins, can promote flowering after being induced and transcribed by FAC [34,35]. *AP1* and *LFY* interact and control flower development during Arabidopsis floral initiation, and the early expression of *AP1* is also induced by *LFY* [14,15]. The expression level of *VcAP1* in the flower buds of blueberry was significantly induced by low-temperature treatment [22]. At present, it has been proved that litchi flowering is induced by low temperature. In the early stage of litchi flowering, *FT* and *LFY* are affected by low temperature. Two *FT* homologous genes were isolated from litchi, namely *LcFT1* and *LcFT2*, and low temperature could only induce the expression of *LcFT1*. The expression level of *LFY* increased after low-temperature induction in litchi at the bud differentiation stage, and gradually decreased with the end of bud differentiation [36,37]. In this study, the expressions of *LcAP1-1* and *LcAP1-2* responded to low temperature, and the expression levels of *LcAP1-2* were significantly induced by low temperature conditions, with minimal expression detected before low temperature and a rapid increase after low temperature treatment. Analysis of the expression patterns of *AP1* showed that *RcAP1* isolated from ‘Old Blush’ of *Rosa chinensis* was expressed in the sepals and leaves, but not in other floral organs [38]. Both mango *MiAP1-1* and *MiAP1-2* genes were strongly expressed in floral organs and weakly expressed in mature leaves and stems [27]. Organ-specific expression analysis showed that *LcAP1-1* was strongly expressed in leaves; however, the high expression level of *LcAP1-2* was observed in flower buds. These data suggest that the *LcAP1-2* gene might be involved in the development of litchi flower buds.

Numerous studies have shown that overexpression of *AP1* genes in *Arabidopsis* induced early flowering in transgenic plants [23,24,26,27]. Similar results were observed in transgenic tobacco overexpressing *PsnAP1*, *MiAP1-1*, and *MiAP1-2* [25,27]. The overexpression of *CsAP1* in ‘Duncan’ grapefruit can also induce blossom early and premature fruit [28]. In this study, we found that the flowering time of *LcAP1-2*-transgenic tobacco was obviously early compared to the wild type, differing from overexpressed *LcAP1-1*. These results suggested that *LcAP1-2* might be involved in the regulation of the litchi flowering process. Interestingly, the transgenic tobacco plants that overexpressed *LcAP1-2* were obviously dwarf compared with the wild type. Similar results have been reported in other species. Overexpression of the *AP1* gene of other species in tobacco will lead to the dwarfing of plants [39]. This may be due to the fact that the transgenic tobacco overexpressing *LcAP1-2* promotes reproductive growth, thereby inhibiting vegetative growth and ultimately affecting plant height. However, there were no significant differences in plant height and flowering time between the transgenic tobacco overexpressing *LcAP1-1* and the wild type. The above results indicate that *LcAP1-1* and *LcAP1-2* have different functions, and that the *LcAP1-2* gene may play a dominant role in the floral transition and flower organ development of litchi, thereby regulating the flowering time of litchi. Precision molecular design breeding is the direction for future development. This study provides an important target gene for the development of litchi varieties with different ripening periods. In the future, the promoter or coding region of the *LcAP1-2* gene in litchi could be edited to delay flowering time, thereby creating late-maturing litchi varieties, which will provide varietal support for the optimization of litchi cultivation structure. Therefore, our research has important applicative value.

## 4. Materials and Methods

### 4.1. Plant Material

The material of ‘Sanyuehong’ was collected from the litchi resource nursery of Guangxi University which has sufficient sunlight throughout the year, an average annual temperature of 21.6 °C, and an average annual rainfall of 1304.2 mm. The materials used in the experiment were all from the six-year-old ‘Sanyuehong’ litchi tree. The seeds of ‘Yunyan 97′ tobacco and *Nicotiana benthamiana* were stored in the Guangxi Crop Genetic Improvement and Biotechnology Lab.

Mature stems, young stems, mature leaves, petioles, pedicel, and flower buds were collected during the floral induction period of ‘Sanyuehong’ litchi. Mature stems are two-year old branches, young stems are 2-month-old branches, leaves and petioles are those harvested 2 months after leaf bud growth, and pedicel and flower bud tissues are from one month after flower bud growth. The flower bud differentiation period for the ‘Sanyuehong’ litchi occurs from approximately November of the previous year to January of the following year. Sampling is conducted based on changes in the morphological characteristics of the terminal or floral buds, with a sampling period from before flower bud differentiation to the formation of flower organs. The samples collected above were subjected to triplicate biological replicates and subsequently stored at −80 °C after cooling with liquid nitrogen for cloning of the *LcAP1* gene and real-time fluorescence quantitative PCR analysis.

### 4.2. Cloning of LcAP1 Gene

In this study, total RNA was extracted from the leaves, stems, and flower buds of all tissue samples using an RNAprep Pure Plant Kit (Tiangen, Beijing, China) and cDNA was synthesized using reverse transcriptase M-MLV (Takara, Kusatsu City, Japan). Two litchi *AP1* homologous genes were isolated, named *LcAP1-1* and *LcAP1-2*. The primers used to isolate *LcAP1-1* were *KLLcAP1-1_F1* and *KLLcAP1-1_R1*, and those used to isolate *LcAP1-2* were *KLLcAP1-2_F1* and *KLLcAP1-2_R1.* The primer sequences are shown in Table 2. Bioinformatics analysis of gene sequences was performed. *AP1* homologous genes from other species were downloaded from the NCBI public database using BLASTx. Clustal W software was used for protein amino acid sequence alignment [40]. The MEGA11 proximity algorithm was used to construct the phylogenetic tree. The protein motifs of two homologous sequences of LcAP1 were analyzed using the online database MEME. The physical and chemical properties of proteins were analyzed using the online database ProtScale. Interacting proteins were predicted using the String online database.

### 4.3. Quantitative Real-Time PCR (qRT-PCR) Analysis

qRT-PCR experiments were conducted using SYBR Premix Ex Taq (TaKaRa, Dalian, China) and a LightCycler 480 system (Roche, Basel, Switzerland). Primers *DLLcAP1-1*_F1 and *DLLcAP1-1*_R1, and primers *DLLcAP1-2*_F1 and *DLLcAP1-2*_R1 were employed to assess the expression level of *LcAP1-1* and *LcAP1-2* mRNA, and the primer sequences are shown in Table 2. The *LcActin* (HQ615689) gene in litchi was used as the internal reference gene for normalizing mRNA levels. The primer sequences for *Lcactin*_F1 and *Lcactin*_R1 are shown in Table 2. Quantitative variation was analyzed using the formula 2^−ΔΔCT^ [41].

### 4.4. Overexpression of LcAP1-1 and LcAP1-2 Genes in Tobacco

In this study, the overexpression vector pBI121-LcAP1 was constructed driven by the CaMV35S promoter and contains nopaline synthase (NOS) terminator sequences. *LcAP1-1* and *LcAP1-2* were insert into the pBI121 vector using an In-Fusion^®^ HD Cloning Kit (Takara, Kusatsu City, Japan), respectively. The constructed vectors were subsequently converted into the bacterial strain Agrobacterium EHA105 [42].

The genes were transferred into tobacco through the tissue culture mediated by Agrobacterium transformation. Transgenic tobacco of overexpressed *LcAP1-1* and *LcAP1-2* were detected by PCR using the primers of 35S_F and *KLLcAP1-1*_R1, 35S_F, and *KLLcAP1-2*_R1. The primer sequences are shown in Table 2. The wild type (WT) was used as the control. The primer sequence is shown in Table 2. The transgenic tobacco was cultivated in a controlled environment chamber of 26 ± 2 °C with a photoperiod of 16/8 h and humidity of 60% to 70%. Three independent transgenic tobacco lines were established, with three technical replicates per line for phenotypic data analysis. The first flowering time is determined as (date of first flower opening)–(sowing date), expressed in days. Concurrently, plant height and leaf number are measured at the onset of flowering.

## 5. Conclusions

The results of this study demonstrated the ubiquitous expression of *LcAP1-1* and *LcAP1-2* in all tissues of litchi. Notably, the expression level of *LcAP1-2* displayed sensitive responsiveness to low-temperature induction. Simultaneously, the expression of the *LcAP1-1* and *LcAP1-2* genes exhibited a strong correlation with the initiation of floral buds and the development of flower organs. However, the functional disparity between the two genes was demonstrated through transgenic tobacco, with the *LcAP1-2* gene exhibiting a capacity to induce early flowering in transgenic tobacco, while *LcAP1-1* does not possess this ability. Therefore, we hypothesized that the *LcAP1-2* gene is involved in regulating litchi flowering rather than *LcAP1-1*. Subsequent research can further explore the regulatory network of *LcAP1-2* in lychee flowering, using *LcAP1-2* as a targeted gene for molecular breeding, and utilizing CRISPR/Cas9 gene editing technology to delay lychee flowering time and obtain late-maturing varieties, thus solving the problem of excessive concentration in lychee fruit production.

## Figures and Tables

**Table 1:** Flowering phenotype data of transgenic tobacco.

Plant Traits at Flowering	WT	OE-*LcAP1-1*	OE-*LcAP1-2*
The time of the first flower (day)	135 ± 2.6	132 ± 1.5	80 ± 1.3 **
Plant height at flowering (cm)	82 ± 3.1	78 ± 2.4	42 ± 2.2 **
Number of leaves at flowering	24 ± 0.8	22 ± 0.6	14 ± 1.2 **

Note: Data are means from three biological replicates ± SE (*n* = 3). ** indicates significance at *p* < 0.01.

**Table 2:** Primers used in this study.

Primer ID	Sequences 5′-3′
*KLLcAP1-1_F1*	GGTGAAGGAAAAGGAGAAGACA
*KLLcAP1-1_R1*	CCCTAAAAGAATAGTGGACGAGTT
*KLLcAP1-2_F1*	GCAGCAACAACAACAACTACCT
*KLLcAP1-2_R1*	CCACAAATGGAAAACCTGAAGA
*Lcactin_F1*	ACCGTATGAGCAAGGAAATCACTG
*Lcactin_R1*	TCGTCGTACTCACCCTTTGAAATC
*DLLcAP1-1_F2*	CACGGGGGACTCTAGAATGGGGAGAGGTCGGGTGCAGCT
*DLLcAP1-1_R2*	AGGGACTGACCACCCGGGTTATTCGTTAAGGTTGCGAAGCA
*DLLcAP1-2_F2*	CACGGGGGACTCTAGAATGGGGAGAGGTAGGGTTCAGTT
*DLLcAP1-2_R2*	AGGGACTGACCACCCGGGTCATGTAGCGAAACATCCAAGAT
*35S_F*	GCACAATCCCACTATCCTTCG

## Data Availability

Data will be made available on request.
